# Tyrosine kinase inhibitors (TKIs) for ovarian cancer treatment: from organic to inorganic chemotherapeutics towards selectivity—a perspective overview

**DOI:** 10.1007/s10534-023-00547-0

**Published:** 2023-11-06

**Authors:** Emma Baglini, Lorenzo Chiaverini, Iogann Tolbatov, Sabrina Taliani, Federico Da Settimo, Diego La Mendola, Elisabetta Barresi, Tiziano Marzo

**Affiliations:** 1 Department of Pharmacy, University of Pisa, Via Bonanno Pisano 6, 56126 Pisa, Italy; 2 Department of Physics and Astronomy, University of Padova, via F. Marzolo 8, 35131 Padua, Italy

**Keywords:** Ovarian cancer, Kinase inhibitors, Inorganic drugs, Metals, Taxanes, Platinum

## Abstract

Ovarian cancer (OC) is a lethal gynecologic cancer in industrialized countries. Treatments for OC include the surgical removal and chemotherapy. In the last decades, improvements have been made in the surgery technologies, drug combinations and administration protocols, and in diagnosis. However, mortality from OC is still high owing to recurrences and insurgence of drug resistance. Accordingly, it is urgent the development of novel agents capable to effectively target OC. In this respect, tyrosine kinase inhibitors (TKIs) may play an important role. Most of TKIs developed and tested so far are organic. However, owing to their chemical versatility, also metals can be exploited to design selective and potent TKIs. We provide a short and easy-to-read overview on the main organic TKIs with a summary of those that entered clinical trials. Additionally, we describe the potential of metal-based TKIs, focusing on this overlooked family of compounds that may significantly contribute towards the concept of precision-medicine.

## Introduction

Ovarian cancer (OC) is the second most common cause of death among gynecological malignancies, and approximately 140,000 women die globally each year from OC (Momenimovahed et al. 2019). This cancer presents subtly, and when diagnosed, treatment options are limited. Conventional first line treatment is the surgical removal of the tumor. After the cancer is removed to the extent possible, there is still a risk that cancer cells remain and may return or spread to other parts of the body. Consequently, chemotherapy is given after, and sometimes before, surgery to destroy these cells, thus improving the chance that the cancer will not recur and decreasing the risk of bad prognosis (Motohara et al. 2018).

**Fig. 1:**
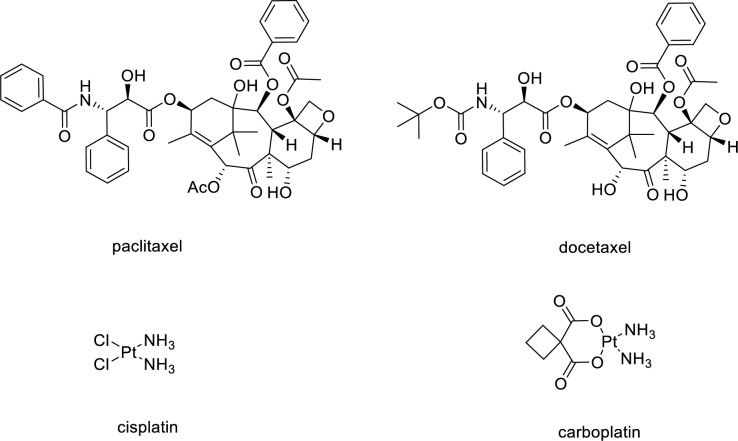
Chemical structures of the reference chemotherapeutics in OC treatment

About 90% of OCs have epithelial origin and are thus called epithelial ovarian cancers (EOC). There are several ovarian cancer subtypes, with up to 80% of patients diagnosed with an EOC subtype of ovarian high-grade serous carcinoma (HGSC). Among the chemotherapeutics, the agents most used in the treatment of OC are taxanes (paclitaxel or docetaxel, Fig. 1) and platinum agents (carboplatin, an analogue of the parent drug cisplatin, Fig. 1), mainly with a combinatory approach.

The commonly recognized mechanism for the anticancer action of cisplatin and carboplatin relies on their ability to bind to the DNA strand hindering both DNA replication and RNA translation, and eventually triggering apoptosis (Siddik 2003; Stordal et al. 2007).

Docetaxel and paclitaxel belong to the class of taxanes, whose anticancer mechanism consists in binding to tubulin dimers, stabilizing the microtubules, thus preventing chromosome movement, and arresting cell division, leading to cell death (Dumontet and Sikic 1999).

Studies have demonstrated that platinum- and taxane-containing chemotherapy improves the survival of women with OC over other types of regimens. As a result, the combination of a platinum-based drug (usually carboplatin) and a taxane (usually paclitaxel) is the standard chemotherapy regimen. Docetaxel and paclitaxel have demonstrated almost identical progression-free survival and overall survival rates in OC, when administered with a platinum-based drug (Katsumata 2003).

Although more than 80% of the patients initially respond to first-line chemotherapy, most of them relapse and drug-resistance and metastasis within 2 years are common events (Ozols 2006). Considering the inherent limitation emerged in the OC treatment, efforts have been devoted towards the development of clinical protocols with agents capable of targeting multiple pathways, including angiogenesis. Accordingly, in the last decade the therapeutic options in the first-line treatments of advanced OC have been implemented by the combination of carboplatin/paclitaxel with the anti-vascular endothelial growth factor (VEGF) antibody bevacizumab and with the recent addition of the poly ADP-ribose polymerase (PARP) inhibitor olaparib. Nevertheless, some limitations remain, mainly relating to identifying patients who are eligible -and thus could benefit most- from the treatment with these agents (Colombo et al. 2019; Marzo and La Mendola 2020).

Thus, despite significant progress in the development of effective approach, treatments capable to arrest/prevent the progression of the disease and the onset of metastatization are needed, and the development of new strategies and the exploitation of alternative therapeutic targets represent key challenges. In this context, achieving new targeted therapy for treating chemotherapy-resistant OC proves to be a stinging weapon.

Targeted therapy is a personalized treatment which refers to the use of agents that interfere with the molecular and biochemical pathways that cause the malignant phenotype, including proliferation, angiogenesis, invasion, metastasis, and decreased apoptosis. Such targeted therapy considers differential expression of specific targets in cancer cells when compared with normal epithelial cells, and their connection to tumor genome and transcriptome for establishing identifiable subgroups of patients who are most likely to benefit from a given therapy. Targeted therapies are being evaluated both as single agents and in combination with chemotherapy (Ozols 2006).

### Small molecules as TKIs for the treatment of ovarian cancer

Based on the evidence that several oncogenic kinase-signaling pathways are dysregulated in OCs patients, small molecule tyrosine kinase inhibitors (TKIs) can be exploited as targeted therapy for those patients who fail the first-line chemotherapy, providing a significant improvement in OC outcome, and becoming part of the current oncology armory. In a nutshell, TKIs are central to personalized medicine in oncology, being often prescribed because of the presence of a peculiar oncogenic mutation (Ng et al. 2022). Furthermore, the design and synthesis of TKIs is relatively feasible because of the structural and functional similarity between the different families (Skorda et al. 2022), encouraging the development of small molecule kinase inhibitors, which has impacted the OC treatment. Accordingly, several TKIs targeting specific signaling pathways, namely VEGFR, platelet-derived growth factor receptor (PDGFR), mast/stem cell growth factor receptor (C-Kit), fibroblast growth factor receptor (FGFR), FMS-like tyrosine kinase 3 (Flt-3), epidermal growth factor (EGFR), and Human epidermal growth factor receptor 1 or 2 (HER1/2), are currently in clinical trials (Phases I, II and III) for the OC treatment (Wang and Fu 2016), see Table 1.

**Table 1:** Clinical trials involving TKIs for the treatment of ovarian cancer (from https://clinicaltrials.gov/, accessed on 10 Jun 2023)

Compound	Target^a^	Status^b^	Phase	Aim^c^	Results^d^	ClinicalTrials.gov Identifier
	VEGFR	C(2014)	2	E/T	Y	NCT00390611
	PDGFR	C(2015)	2	E/S	NA	NCT01047891
		T(2016)	1/2	E/X	Y	NCT00526799
		C(2015)	2	E	Y	NCT00791778
		C(2013)	2	E	NA	NCT00096395
		C(2020)	2	E	Y	NCT00436215
		T(2016)	2	E/S/X	Y	NCT00522301
		C(2019)	2	E	NA	NCT00093626
		C(2022)	2	E	Y	NCT00096200
	VEGFR	C(2018)	2	E	NA	NCT01824615
	PDGFR	C(2020)	2	E/T	Y	NCT00979992
	C-kit	C(2018)	2	E	Y	NCT00768144
		C(2015)	2	E/T	Y	NCT00388037
		C(2014)	2	E	NA	NCT00543049
		C(2020)	2	E	Y	NCT00478426
	VEGFR	U(2016)	1/2	E/S	NA	NCT01600573
	PDGFR	C(2016)	1/2	E	NA	NCT01238770
		C(2022)	2	E	NA	NCT02383251
		C(2021)	3	E/S	Y	NCT00866697
		T(2021)	1/2	D	NA	NCT02055690
		C(2016)	2	E/T	NA	NCT01262014
		C(2015)	2	E/S	Y	NCT01227928
		C(2018)	2	E/S	NA	NCT01644825
		C(2021)	2	E/S	Y	NCT01610206
		C(2018)	2	E/S	Y	NCT00281632
		C(2023)	1/2	D/T	NA	NCT01402271
		T(2021)	1/2	E/S/T	Y	NCT01035658
		C(2019)	2	E	Y	NCT01468909
		C(2012)	2	S/X	Y	NCT00561795
	VEGFR	T(2014)	1	D	Y	NCT01329549
	PDGFR	T(2022)	1	S	NA	NCT01485874
	FGFR	C(2019)	2	E/S	NA	NCT01610869
	C-kit	C(2018)	2	E	Y	NCT01669798
	Flt-3	C(2023)	2	PFS	NA	NCT01583322
		C(2016)	2	PFS	Y	NCT00710762
		C(2018)	1	D	Y	NCT01314105
		C(2017)	3	E	Y	NCT01015118
		U(2016)	2	E	NA	NCT02866370
	VEGFR	C(2021)	2	E	Y	NCT01853644
		T(2017)	2	E	Y	NCT01972516
	EGFR	C(2022)	1/2	D/E	Y	NCT00317772
		C(2013)	2	E/T	NA	NCT00023699
		C(2012)	2	E	NA	NCT00181688
		C(2010)	2	S/X	NA	NCT00189358
		C(2013)	2	E	NA	NCT00049556
	VEGFR	C(2018)	2	E	NA	NCT02340611
	C-kit	U(2015)	3	S/E	Z	NCT00532194
		C(2022)	NAP^e^	E	NA	NCT02681237
		C(2022)	2	E/S	Y	NCT02889900
		R(2022)	3	E/S/X	NA	NCT03278717
		A(2022)	2	E	NA	NCT03117933
		U(2019)	2	S/E	Z	NCT03314740
		C(2018)	2	E	Y	NCT00278343
		C(2022)	1	E	NA	NCT02855697
		C(2015)	2	E	Y	NCT00275028
		A(2022)	2	E	Z	NCT02345265
		A(2022)	1/2	D/T	Y	NCT01116648
		A(2023)	3	E	Y	NCT02446600
		A(2023)	2/3	E	NA	NCT02502266
		R(2023)	1/2	E	NA	NCT02484404
		U(2021)	2	E	NA	NCT03699449
		C(2014)	1	T/D	NA	NCT01131234
		C(2021)	1	S	NA	NCT01065662
	EGFR	T(2016)	2	E	Y	NCT01003938
		C(2012)	2	E/S	Y	NCT00130520
		C(2013)	3	E/S	NA	NCT00263822
		C(2010)	1/2	T/D	NA	NCT00217529
		C(2020)	2	E	NA	NCT00030446
		C(2014)	2	E/T	NA	NCT00126542
		C(2015)	2	T	Y	NCT00059787
		C(2018)	2	PFS/T	Y	NCT00520013
	EGFR	R(2023)	1	D	NA	NCT04608409
	HER2	T(2009)	1	D	NA	NCT00317434
		C(2019)	2	E	Y	NCT00113373
		C(2014)	2	E	Y	NCT00436644
		C(2011)	1/2	E/S/X/D	NA	NCT00316407
		T(2009)	2	E	NA	NCT00888810

^*a*^*EGFR* epidermal growth factor receptor, *FGFR* fibroblast growth factor receptor, *PDGFR* platelet-derived growth factor receptor, *VEGFR* vascular endothelial growth factor receptor, *C-kit* mast/stem cell growth factor receptor, *Flt-3* FMS-like tyrosine kinase 3

^b^A = Active, not recruiting, C = Completed, R = Recruiting, T = terminated, U = Unknown, the year of the last update is reported in brackets

^c^D = Dosage; E = Efficacy; P = Pharmacokinetic; PFS = progression free survival; S = Safety; T = Toxicity; X = Tolerability

^d^Y = Results published; Z = Results submitted; NA = Results not available

^e^NAP = Not applicable

Promising results against OC were obtained using TKIs targeting angiogenesis, one of the hallmarks of cancer biology; indeed, by means of this process, tumors create a network of vessels useful for gas exchange, nutrient supply, and elimination of waste substances. In light of the above, the development of TKIs able of interfering between the binding of VEGF to its receptor, VEGFR (Baert et al. 2021), may represent an efficient method for fighting the tumor growth and invasion in many cancers, including OC. Other promising TKIs developed for the treatment of OC include agents targeting growth factors as FGF, PDGF and EGF. These interactions block the intracellular signaling cascades, which would otherwise be caused by the binding to their receptors and ensure cancer arrest.

Some of the clinical trials involving TKIs are summarized in Table 1 with the main details.

### Looking beyond organic entities: the case of metal complexes

**Fig. 2:**
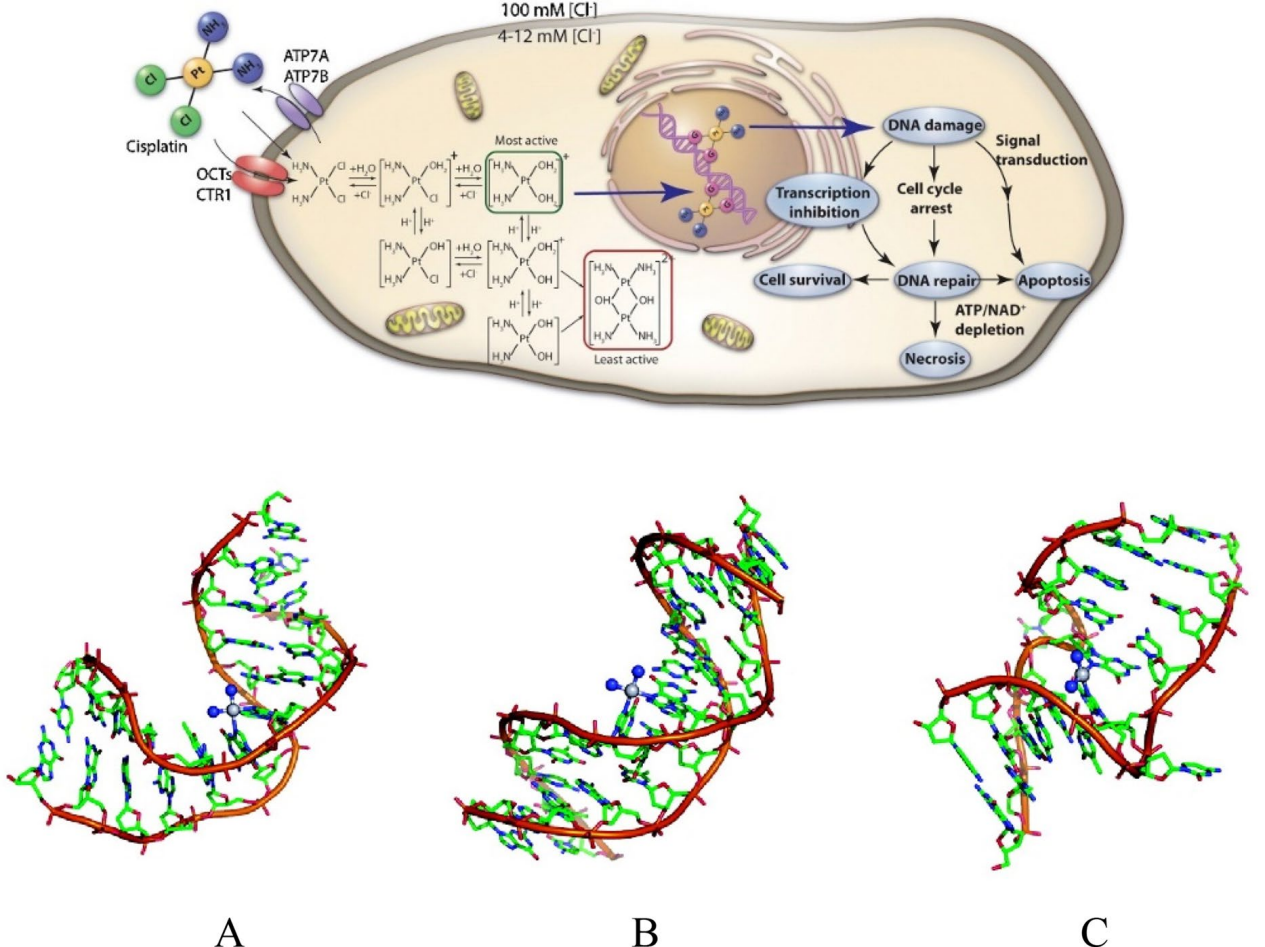
The mechanism of cisplatin activation. Cisplatin is internalized into the cancer cells by both passive diffusion and via copper transporter 1 (Ctr1) mediated transportation. After internalization, owing to the decrease of chloride concentration from outside the cells (⁓100 mM) to within the cancer cells (⁓4–12 mM), cisplatin undergoes aquation to mono or bis aquo-species. These latter are capable of binding towards nuclear DNA, blocking replication with induction of apoptosis (top) [reproduced and adapted with permission from reference (Ma et al. 2015)]. Platinum–DNA adduct structures. Duplex DNA containing **A** cisplatin 1,2-d(GpG), **B** 1,3-d(GpTpG) intrastrand, and **C** interstrand cross-links (bottom) [reproduced and adapted with permission from reference (Jung and Lippard 2007)]

Metallodrugs are molecules featured by the presence of a metal center coordinated by ligands. In these chemical entities, the metal generally acts as functional center -thus drives the specific reactivity toward biological substrates- while the ligands -that can be characterized by selected properties- may be designed to impart the desired characteristics to the molecule, thus allowing the modulation of chemically relevant parameters such as stability, lipophilicity and propensity of the coordinated metal center to react under specific conditions (Messori et al. 2014b; Anthony et al. 2020; Cirri et al. 2022). Owing to their versatility, inorganic molecules are widely applied in medicine for several purposes and with a role that is not replaceable by organic molecules. For instance, Gd(III) compounds are essential as relaxing agents for the improvement of the quality of the Magnetic Resonance Imaging (MRI) data, and consequently for the diagnosis of pathological conditions, including cancer. Also radiopharmaceuticals for cancer often contain metals. Examples are ^99m^Tc, ^68^ Ga, ^64^Cu complexes that find extensive application in single photon emission computed tomography (SPECT) or positron emission tomography (PET) imaging techniques; while [^223^Ra]RaCl_2_ dichloride treatment was approved by FDA in 2013 to treat patients with metastatic castration-resistant prostate cancer (mCRPC) because of the associated improvement of the overall survival (Specht and Berthelsen 2018; Sraieb et al. 2020). However, the reference inorganic drug in cancer treatment is undoubtedly cisplatin. This Pt(II)-containing compound was approved by FDA in 1978 and since it has revolutionized the clinical approach in several kind of cancer including ovarian, testicular, head-neck and others. Together with its second and third generation analogues carboplatin and oxaliplatin (approved respectively in 1989 and 2002), they are used in almost 50% of anticancer chemotherapy clinical protocols (Fig. 1) (Ma et al. 2015; Zhang et al. 2022b; Cirri et al. 2022). Basically, the main mechanism for the anticancer activity of cisplatin relies on its activation after intravenous administration (Fig. 2).

Indeed, is the drop of chloride concentration inside the cells that induces the release of the ligands, and the subsequent formation of the aquo-active species capable of DNA coordination. This is a substantially non-specific mechanism for the induction of antitumor effects implying the well-known limits of the platinum-based chemotherapeutics (Oun et al. 2018). In this view, carboplatin, i.e. the reference platinum molecule for OC treatment, share with cisplatin a similar profile of activation as well as a similar effectiveness, but has lower toxicity for patients substantially attributable to the nature of the ancillary ligand. In fact, at variance with cisplatin, carboplatin, once injected also undergoes activation, but in this case, it relies on the release of the cyclobutane-dicarboxylic acid (CBD) ligands (see Fig. 1 for structural details) (Marzo and Messori 2023). In turn, the small, but still relevant differences in the chemical structures, determine a slower activation kinetic -and accordingly a decreased reactivity- likely concurring to the decreased side effects of carboplatin with respect to cisplatin (Messori et al. 2015; Marzo and Messori 2023). From this comparison it emerges as the use of specific ligands at the functional metal center may dramatically impact the overall pharmacological profile of metallodrugs. Indeed, it is possible to modulate the therapeutic outcome through the proper choice of the metal ligands. For instance, it is possible to stabilize a metal center in a specific oxidation state, regulate its reactivity based on the administration route or using stimuli-sensitive ligands that trigger the activation of the compound only under specific conditions (e.g. at the tumor site owing to the lower pH) (Xu et al. 2014; Qu et al. 2017; Canil et al. 2019; Barresi et al. 2020; Liu et al. 2021; Wootten et al. 2022; Zhang et al. 2022a). In the next paragraphs, we will highlight as the chance of preparing compounds featured by high stability, together with the possibility of imparting selected geometries through the accurate selection of ligands, represent the key aspects for the development of selective inorganic TKIs.

### Established inorganic drugs affect tyrosine kinase activity: the effects on angiogenesis: some examples

**Fig. 3:**
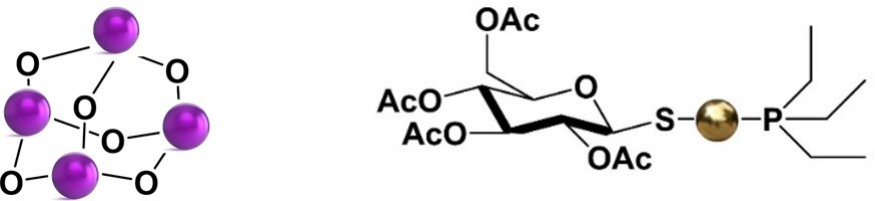
Chemical structures of (from left) As_2_O_3_ (trisenox) and auranofin (the purple and gold spheres stand for As and Au, respectively)

Angiogenesis represents a key process for the spreading and the progression of OC. Bevacizumab (marked under the brand name Avastin®), is a humanized anti-VEGF monoclonal antibody, that positively impacted the OC treatment and management since the approval by FDA and other regulatory agencies worldwide. Despite the current role of the approved agents targeting angiogenesis for the clinical management of OC, yet several limitations do exist to their clinical use, including the lack of the availability of biomarkers capable to predict their precise efficacy in different patients so that to limit adverse effects and improve the prognosis (Collinson et al. 2013; Secord et al. 2020). In accordance with this, several small-molecule antiangiogenic agents, including VEGFR TKIs have been developed, but efforts led -to date- to controversial/not fully satisfactory results or to clinical trials that are still ongoing. Additionally, also molecules affecting other targets have been tested (e.g. PARP inhibitors and immune checkpoint inhibitors see Table 1) (Monk et al. 2016). However, a lesser-known aspect is the ability of some established metallodrug to interfere with TK-dependent pathway related with angiogenesis. As matter of fact, metal ions can regulate directly or indirectly VEGF activity both in a positive or negative fashion (Saghiri et al. 2015). It is interesting to remark as this consideration may drive the therapeutical approach or the use of specific drug cocktails for improved OC treatments. This is the case of platinum-based drugs and specifically carboplatin. When the drug is administered as monotherapy, its effect is the stimulation of the overexpression of VEGF, likely concurring to the survival of endothelial cells, which, in turn, acts as protection from the anticancer activity of the drug itself. Hence, the combination of carboplatin treatment with anti-VEGF agents is a reliable strategy in OC capable to significantly potentiate the outcome of platinum-based protocol (Marzo and La Mendola 2020). Beyond carboplatin, even As_2_O_3_ has some effects on VEGF driven mechanisms. It is a simple As(III)-based compound (Fig. 3), known since centuries in the Chinese traditional medicine and approved in 2000 by FDA under the brand name Trisenox® for treating promyelocytic leukemia (APL). It seems to have a two-faced activity. Indeed, it induces an augmented VEGF expression in OVCAR-3 human OC cells, whereas this effect is absent in leukemia cells. Also, it has been highlighted as the administered dose is a key aspect for triggering either effects (Roboz et al. 2000; Duyndam et al. 2003). Auranofin (brand name Ridaura®) is an FDA approved drug orally administered in the treatment of rheumatoid arthritis, that has been recently repurposed as anticancer agents against various tumors, including OC (Fig. 3). In this frame, it entered some clinical trials (see clinicalTrials.gov website) (Massai et al. 2022). Interestingly, it was reported as auranofin is capable of direct inhibition of VEGFR-2 phosphorylation, thus impairing angiogenesis because of the blockage of VEGFR-2 activation (Koch et al. 1988; He et al. 2014). In 2014, Chen et al. found out as auranofin targets vascular endothelial growth factor receptor-3 (VEGFR3), an endothelial cell (EC) surface receptor essential for angiogenesis and lymphangiogenesis (Chen et al. 2014). Interestingly, auranofin blocks VEGFR3 in a dose-dependent manner.

Mechanistically speaking, auranofin, for higher doses, decreases the level of the protein thioredoxin reductase (TrxR2, that as a key role in cellular survival), TrxR2-dependent Trx2 and transcription factor NF-B, concomitantly increasing the stress signaling p38MAPK, leading to ECs apoptosis. At variance, if administered for lower doses, auranofin induces downregulation of VEGFR3 and VEGFR3-mediated EC proliferation and migration, two important steps for in vivo lymphangiogenesis. Also, auranofin-induced VEGFR3 downregulation is blocked by *N*-acetyl-l-cysteine as well as by chloroquine (a lysosome inhibitor), but it is promoted by proteasomal inhibitors (MG132). Authors conclude that auranofin induces VEGFR3 degradation through a lysosome-dependent pathway (Chen et al. 2014). Overall, it seems that TK-dependent pro- or anti-angiogenic effects of these complexes are substantially related not only on the tumor’s features, but also on the specific administration protocols, in terms of both timing and amount. From this information we can draw some interesting hints. For instance, as the antiangiogenic effects of auranofin do not rely on the TrxR system inhibition; it could be envisioned an evaluation of auranofin for a double dose-dependent use. Indeed, for non-toxic doses, it might act as antiangiogenic agent, whereas for higher doses, it might act as cytotoxic agent affecting the redox homeostasis of cells through interaction through its binding towards TrxR system (Marzo and La Mendola 2020).

### Tailored metal-based drugs as selective TKIs

**Fig. 4:**
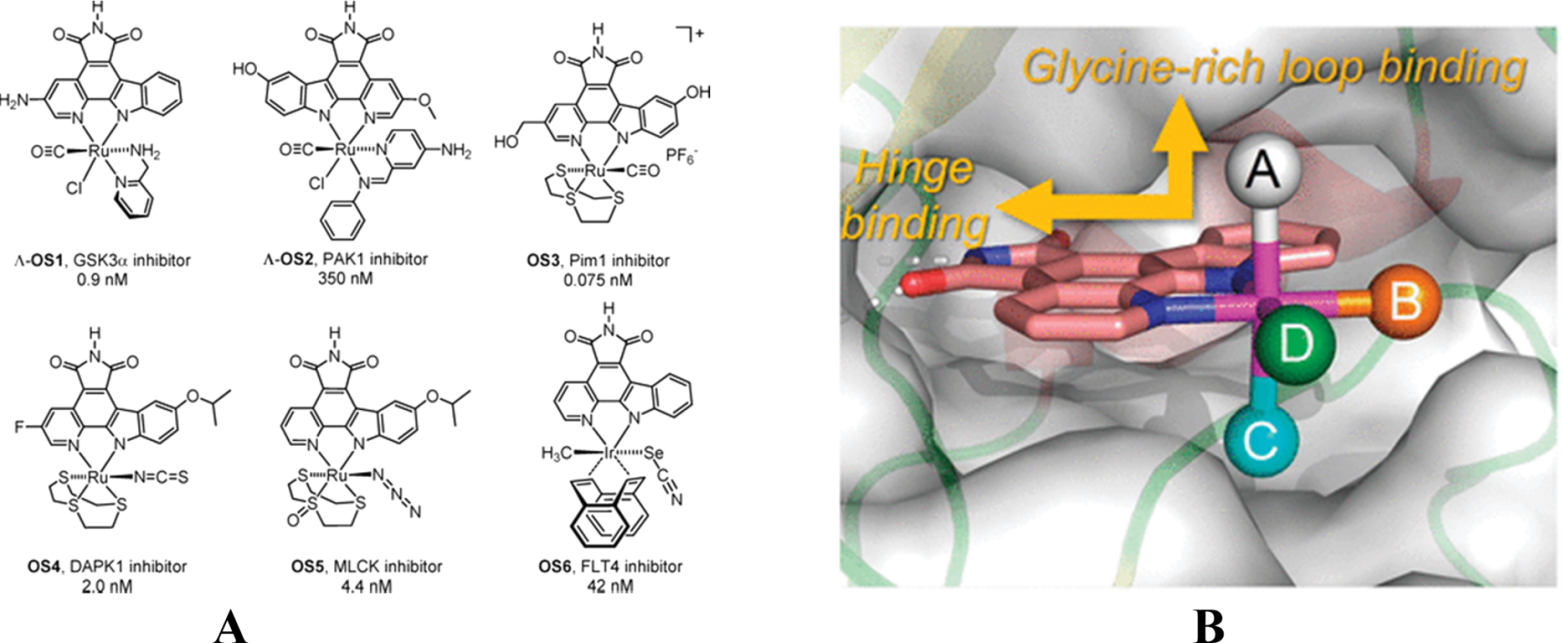
Chemical structures of the six complexes developed by Meggers et al. these are highly selective octasporine protein kinase inhibitors. The reported IC_50_ values (nM) were determined in the presence of 100 μM ATP **A**. Binding of the octahedral pyridocarbazole metal complex scaffold to the ATP-binding site of a protein kinase. The metal center in combination with the coordinating ligands (A–D) controls the shape and functional group presentation of the molecular scaffold. A unique aspect of this octahedral scaffold is that the orthogonal orientation of the pyridocarbazole heterocycle and the ligand A simultaneously enables efficient interactions with both the hinge region and the glycine-rich loop **B**. Reproduced and adapted with permission from reference (Feng et al. 2011)

The cisplatin mode of action described above has represented the paradigm for metallodrugs anticancer activity for several years (Dasari and Bernard Tchounwou 2014). However, to date, it is ascertained as metallodrugs are capable of tight interaction towards several biological substrates including proteins (Messori et al. 2014b; Merlino et al. 2017; Salerno et al. 2021). The coordination of metallodrugs to protein has a huge impact in terms of pharmacological effects. For instance, cisplatin, once intravenously administered, quickly reacts with serum proteins, including the most abundant human serum albumin (HSA) (Massai et al. 2019). The effects of the binding significantly impact the overall anticancer effects as well as the insurgence of side effects (Massai et al. 2019; Sarpong-Kumankomah and Gailer 2020). The protein-metalation process may occur via multiple mode, including covalent binding or non-covalent interaction (Messori et al. 2014a; Martín-Santos et al. 2015; Marzo et al. 2022). This is the consequence of the design of inorganic drugs that substantially act as prodrugs, undergoing activation after the selective release of ligands under specific conditions. However, it should be remembered that the high versatility of inorganic compounds may be conveniently exploited for the preparation of complexes endowed with unique stability and geometries. These features are of paramount importance when we aim to develop metal-based compounds featured chemical structures with natural-product-like shape and unique stereochemical complexity. In other words, inorganic medicinal chemistry, and inorganic compounds, offer unique opportunities that are not reproducible by organic molecules, being these latter not easily exploitable to build 3D complexity. These opportunities mainly rely on the chance of preparing metal-based complexes endowed with unprecedented specificity for the selective inhibition of protein kinases (Feng et al. 2011). Noticeably, a few years ago Meggers and co-workers published a seminal paper reporting the proof of concept that carefully tailored inorganic compounds may offer the degree of sophistication to succeed in the developments of innovative KIs capable on unprecedented specificity (Feng et al. 2011). These authors report on six metallodrugs bearing ruthenium(II) or iridium(III) metal centers (Fig. 4).

Remarkably, each of this metal compounds is characterized by high selectivity for an individual protein kinase i.e., GSK3R, PAK1, PIM1, DAPK1, MLCK, and FLT4 (Feng et al. 2011). Basically, the developed compounds were conventional ATP-competitive inhibitors, however the combination of the unconventional globular shape and other characteristics including the rigidity, make them highly selective TKIs with octahedral coordination. This latter feature allows the specific interaction towards glycine-rich loop, significantly contributing to the unique characteristics of these TKIs. The impact that development, implementation, and preclinical testing of these compounds may have in OC treatment is well witnessed by inspection of the activity of compound OS6. It is capable of selective inhibition of the receptor tyrosine kinase FLT4 (also known as VEGFR3), being this latter crucial in OC proliferation (Su et al. 2006; Sopo et al. 2019). Alongside, other metal-based strategies for improved TKIs development have been investigated, including the synthesis and testing of Metal-TKIs conjugates. This with the aim to resolve the current limitation in overcoming acquired and innate resistance as well as dose-limiting side effects of purely organic compounds, such as Erlotinib and Sunitinib.

**Fig. 5:**
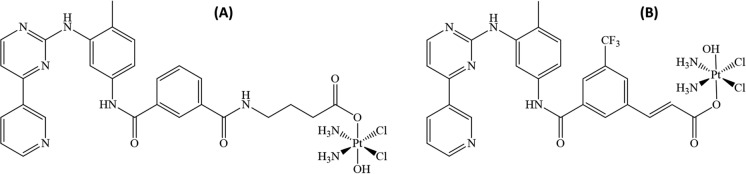
The Pt(IV) anticancer pro-drugs based on cisplatin-like chemical structure and derivatives of the tyrosine kinase inhibitors imatinib (**A**) and nilotinib (**B**)

The idea of combining the effects of anti-tumour organic drugs with the effect of a metallodrug is ubiquitous nowadays and allows to reach the efficacy levels possible only due to the synergistic effect of both pharmaceutical agents. The Pt(IV)-bearing complexes are particularly promising in this regard due to the increased stability, under physiological conditions, of this oxidation state in comparison to the species featured by the presence of Pt(II) center. Moreover, the augmented lipophilicity increases the cellular intake. Being activated in the cell via the reduction of Pt(IV) to Pt(II), this scaffold produces cisplatin inside the cell and two released axial ligands. Recently, two novel Pt(IV) anticancer pro-drugs based on cisplatin-like structures combined with derivative of either imatinib or nilotinib in the axial position were produced (Fig. 5) (Li et al. 2023). The inhibitory capacity of these pro-drugs on three TK targets, PDGFR-α, c-KIT and the T670I mutated c-KIT was predicted computationally and confirmed on the isolated enzymes. Moreover, it was demonstrated the robust activity against OC cell line. Here we should mention that the activity of these drugs was ameliorated with respect to free imatinib and nilotinib due to their increased lipophilicity when coordinated to the metal center.

Overall, some preclinical promising evidences of this approach have indicated the potential of the conjugates to circumvent or control problems related to selectivity, and ameliorate pharmacological properties. Several compounds based on various heavy metals including platinum, cobalt, iron, copper have been prepared, including those reported in 2019 by Qin and coworkers (2019). However, for details on the strategy for the development of metal-based conjugate TKIs, we suggest referring to the beautiful and comprehensive review published in 2022 by Beirne et al. (2022). We have outlined some interesting strategies for developing novel TKIs. These strategies despite the advantages, nevertheless, are based on the use of transition metals for which the likelihood that the central metal ion remains inert in vivo is relatively small. This may imply some side effects that, without carefully designed studies, may hamper the further progress of compounds. In fact, if metal release occurs the presence of the metal may represent in the flip side of the coin. Eventually, it should be also noticed that only and more systematic studies on these aspects may ensure a better understanding towards innovative and unconventional TKIs (Boros et al. 2020).

On the ground of the arguments discussed in this report, it results as beyond the organic molecules that are being developed as specific TKIs for treating OC, inorganic compounds may offer a reliable approach to further expand the chemical space and chemical versatility towards highly specific innovative compounds capable of unprecedented specificity. Pairwise, it should be highlighted as the goal of clinical use of inorganic entities as TKIs in OC is still far away and only deeper studies and larger screening might spur this important opportunity.

### Future perspectives

We have demonstrated that exist a plethora of investigations dedicated to the synthesis and employment of either organic or inorganic small-molecule kinase inhibitors against OC. Nevertheless, the results of trials based on monotherapy utilizing a specific TKI are usually poor, the reason of that being the large diversity of the OC, in particular HGSC tumors. This heterogeneity is the main obstacle that stands behind the necessity to use the therapy based on several particular kinase inhibitors and targeting several signaling routes.

Indeed, the consensus in the field is that the targeting of a particular step in one signaling route is not a sufficient condition for the guaranteed impediment to the downstream events, which could be reignited by a signal from another process. There are two possible strategies to overcome this obstacle: the “horizontal” and “vertical” blockades. The “horizontal” corresponds to the therapy in which there is more than one active TKI and all the inhibitors aim at different pathways, whereas the “vertical” blockade intends to impede several steps of the same pathway (Katopodis et al. 2019).

Moreover, a tumor in every specific case displays various genetic anomalies and expression profiles, thus leading to the imperative of application of personalized therapy strategies including the analysis of patient-derived ex vivo tumor organoid cultures and DNA/RNA sequencing. Here, it is essential to employ the methods not only capable to preserve the organoids from patients but also able to save the original features of tumors ex vivo. The sequencing of these patient-based samples produces a substantial amount of data sufficient for designing efficacious individual therapy strategies. Another possibility is the advancement of patient-derived xenograft models, the major setback here being the prevention of immune attacks against the xenotransplanted tumor. An alternative, ex vivo option is the so-called microfluidic-based cancer-on-a-chip model, where multiple cancers are grown together to analyze the activity of kinase inhibitors in various combinations.

## Data Availability

Not applicable.
